# Comparative analysis of cutaneous features of psoriasis in acute and chronic imiquimod-induced mouse models

**DOI:** 10.1038/s41598-025-12111-6

**Published:** 2025-07-23

**Authors:** Emma Fraillon, Jean-François Jégou, Hanitriniaina Rabeony, Jean-Claude Lecron, Nicolas Lebonvallet, Emilie Marie-Joseph, Audrey Josset-Lamaugarny, Géraldine Aimond, Laurent Misery, Franck Morel, Fabien P. Chevalier, Bérengère Fromy

**Affiliations:** 1 Laboratoire de Biologie Tissulaire et Ingénierie Thérapeutique, CNRS UMR5305, Université Claude Bernard Lyon 1, Lyon, France; 2 Laboratoire Inflammation, Tissus Épithéliaux et Cytokines, Université de Poitiers UR15560, Poitiers, France; 3 Centre Hospitalier et Universitaire (CHU) de Poitiers, Poitiers, France; 4 Laboratoire Interactions Épithéliums Neurones, Univ Brest UR4685, Brest, France; 5 Centre Hospitalier et Universitaire (CHU) de Brest, Brest, France

**Keywords:** Inflammation, Skin diseases, Animal disease models

## Abstract

Psoriasis is a chronic inflammatory skin disorder influenced by genetic and environmental factors. The imiquimod (IMQ)-induced mouse model is widely used to study psoriasis-like skin inflammation. However, its current use is largely limited to short treatment periods. Therefore, this model predominantly reflects acute inflammation and is limited in its capacity to capture the chronic nature of psoriasis. To date, little attention has been given to the extension of IMQ treatment duration to study the characteristics of sustained inflammation. This study aimed to provide the first direct comparison between short-term (ST, 4 days) and long-term (LT, 9 weeks) IMQ treatments on cutaneous histological, molecular and functional psoriasis features and to assess their similarity with human psoriasis. Both models demonstrated similar global severity, but with distinct features: ST-IMQ treatment led to increased epidermal hyperplasia and impaired skin barrier function, while LT-IMQ treatment was associated with greater desquamation and impaired suprabasal differentiation. Dermal mechanical properties and endothelial dysfunction were similar between the two models. Gene expression analysis revealed differentially expressed psoriasis-related markers between ST- and LT-IMQ treatments, with both models showing similarities to human psoriasis. These findings offer critical insights into how acute and chronic models can be tailored to study specific psoriasis features.

**Supplementary Information:**

The online version contains supplementary material available at 10.1038/s41598-025-12111-6.

## Introduction

Psoriasis is a Th17-mediated chronic inflammatory skin disorder influenced by genetic and environmental factors, affecting 0.09–11.3% of the global population^1^. In psoriasis, sustained inflammation results in various cutaneous features, including epidermal hyperplasia with parakeratosis and hyperkeratosis, defective skin barrier function^2,3^ and increased skin rigidity^4^. Psoriatic inflammation also extends beyond the epidermis and dermis^5,6^leading to impaired vascular functions^7^likely due to vascular inflammation indicated by higher basal skin blood flow in psoriasis patients^8^.

Despite effective biotherapies targeting key cytokines such as TNF-α, IL-17 and IL-23^9^, no permanent cure is available for patients. This underscores the need for relevant animal models that accurately reflect the chronicity and systemic inflammation of the disease to facilitate novel therapeutic strategies. Various mouse models of psoriasis exist, including spontaneous mutations in specific genes such as *Scd1* or *Ttc7*^10,11^, xenotransplantation of human psoriatic skin or immune cells in immunodeficient mice^12^and genetic or inducible models^13–17^. Among these, a simple and useful method to induce psoriasiform dermatitis in mice is the topical administration of imiquimod (IMQ), an agonist of Toll-Like Receptor 7/8 (TLR 7/8), which activates plasmacytoid and myeloid dendritic cells^13^. In this model, daily IMQ application on depilated mouse skin for 4 to 7 days reproduces macroscopic (thick and scaly skin) and microscopic (acanthosis, hyperkeratosis, parakeratosis) features of psoriasis, together with the activation of the Th17 signaling pathways (IL-23/IL-17 axis) and the subsequent recruitment of immune cells (e.g. T cells, neutrophils, dendritic cells)^13,14^. The resultant skin lesions are thus developed in a comparable manner to the human disease, directly by initiating the immune cascade.

However, the IMQ-induced psoriasis-like mouse model has limitations, particularly its inability to fully replicate the chronic nature of human disease. To address this, Vasseur et al.^18^ developed a chronic psoriasiform model in 2018, applying IMQ biweekly for nine weeks. They investigated the long-term effects of IMQ treatment on other organs, revealing associations with liver inflammation and fibrosis^18^. However, the cutaneous features of both acute and chronic models remained to be explored.

In this study, we characterized the IMQ-induced psoriasis-like skin at the clinical, histological, molecular and functional levels, including barrier function, mechanical properties and endothelial dysfunction, following two treatment durations: a short-term (4 days) treatment to model acute inflammation and a long-term (9 weeks administered biweekly) treatment to mimic chronic inflammation. We also compared the cutaneous expression of several psoriasis-related genes in short-term (ST) and long-term (LT) IMQ-treated mouse skin with lesional skin from human psoriasis patients to assess their relevance to human psoriasis.

## Results

### Macroscopic cutaneous features differ after short-term and long-term IMQ exposures

**Fig. 1:**
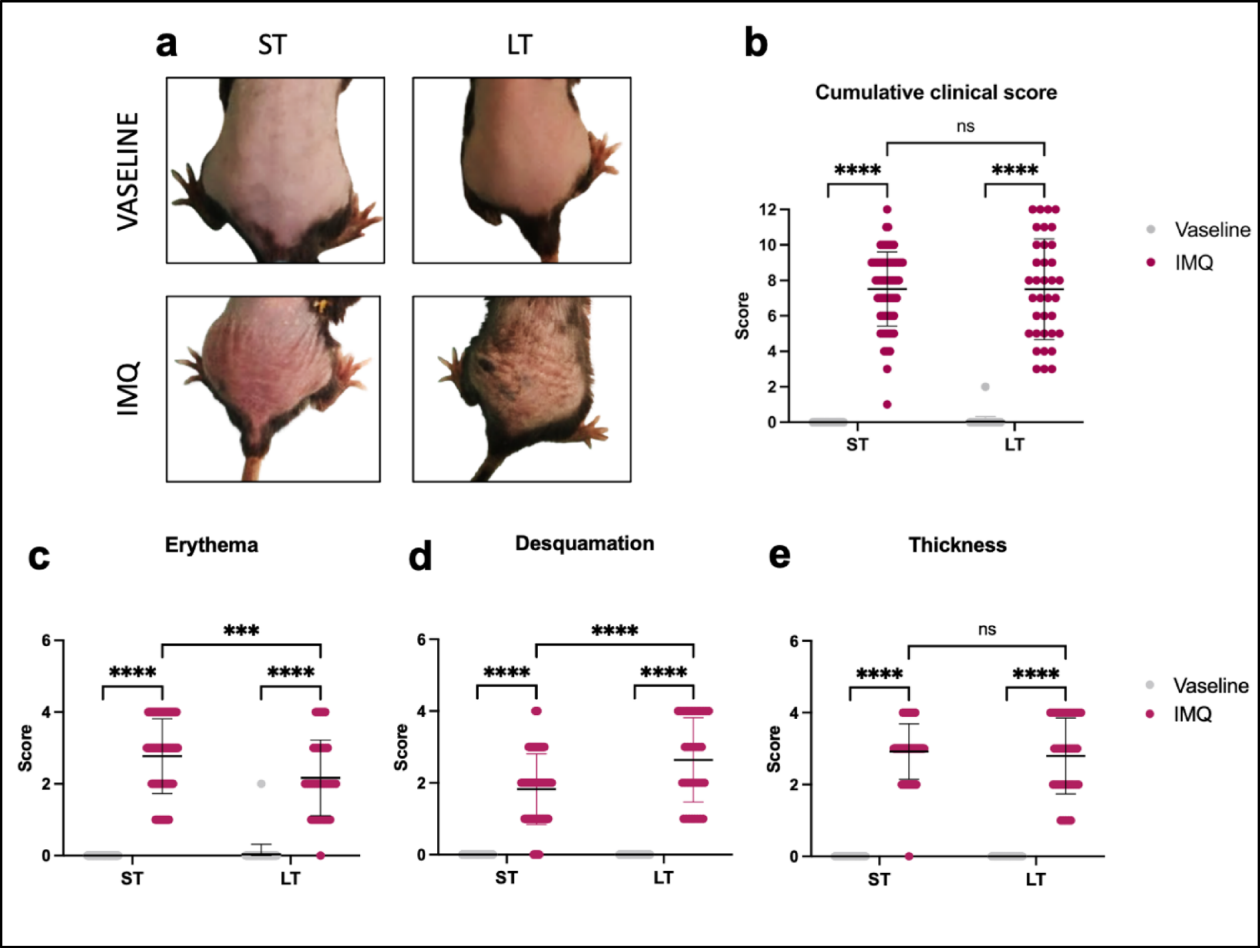
Effects of IMQ on macroscopic and clinical features of mouse skin after short-term (ST) and long-term (LT) treatments. (**A**) Representative photographs of ST and LT treatments-induced macroscopic features on mice back skin. (**B**) Cumulative clinical scores (sum of the scores for erythema (**C**), desquamation (**D**) and thickness (**E**)) after ST and LT treatments with Vaseline or IMQ (ST-Vaseline *n* = 82, ST-IMQ *n* = 77, LT-Vaseline *n* = 66, LT-IMQ *n* = 64). A Two-Way ANOVA with Bonferroni’s correction was performed to compare each parameter after ST and LT treatments either with Vaseline or IMQ.

Both short-term (ST) and long-term (LT) treatments with IMQ induced the appearance of erythema associated with thickened and scaly skin in mice (Fig. 1a). Cumulative clinical scores were similar after both ST (7,5 ± 2,1) and LT (7,5 ± 2,8) IMQ treatments, whereas null values were associated to Vaseline treatments (Fig. 1b). Although no significant difference in the cumulative clinical score was observed between ST and LT-IMQ treatments (Fig. 1b), a stronger erythema was observed after ST-IMQ treatment (2.9 ± 1.4) compared to LT-IMQ treatment (2.2 ± 1.1) (Fig. 1c), whereas desquamation was greater after LT-IMQ treatment (2.6 ± 1.2) compared to ST-IMQ treatment (1.8 ± 1.0) (Fig. 1d). In contrast, no significant difference was observed in skin thickness between the two treatment durations (Fig. 1e). These differences were not linked to differential IMQ concentration in the skin of ST- and LT-IMQ treated mice as we observed no differences in IMQ concentrations in the back skin of mice at the end of both treatments (data not shown).

Interestingly, longitudinal monitoring of the cumulative clinical scores in the LT protocol demonstrates that all parameters dropped drastically during the untreated weeks to come closer to control values, suggesting skin recovery during the untreated weeks of the LT protocol (Supplementary Fig. S1).

In the ST group, IMQ led to a transient weight loss during the first two days of treatment compared to ST-Vaseline-treated mice (Supplementary Fig.S1). This transient weight loss was also observed during every treated weeks (1, 3, 5, 7, 9) of the LT-IMQ treatment, with weight stabilization during untreated weeks (2, 4, 6, 8) (Supplementary Fig. S2).

### IMQ-induced molecular signature differs after short-term and long-term exposures

**Fig. 2:**
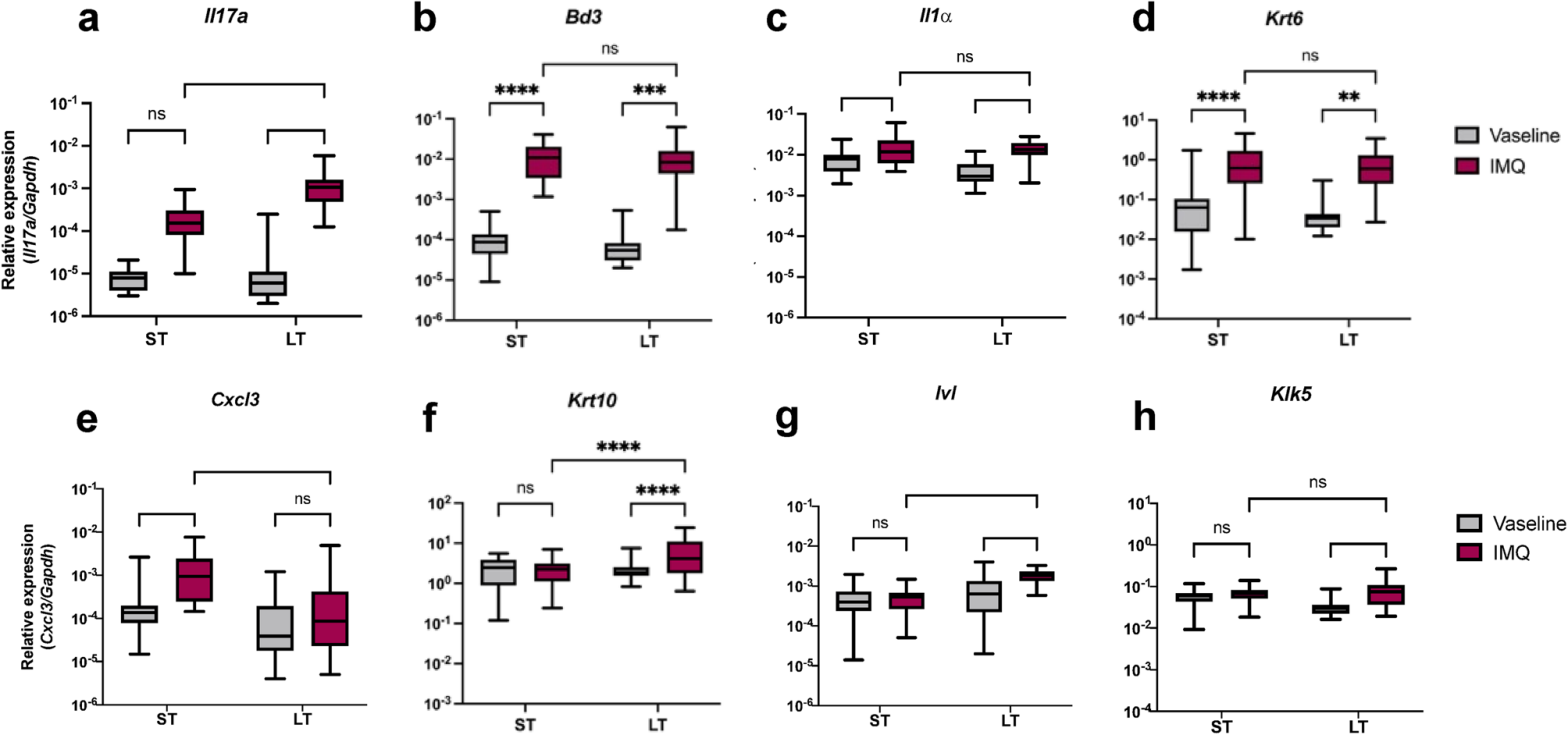
Effects of IMQ on the gene expression levels of pro-inflammatory mediators and epidermal markers after ST and LT treatments. Pro-inflammatory mediators (IL-17A (**A**), IL-1α (**B**), CXCL3 (**C**)), AMPs (BD3 (**D**)) and epidermal markers (KRT6 (**E**), KRT10 (**F**), IVL (**G**), KLK5 (**H**)) gene expression was assessed by RT-qPCR on total mRNAs from mouse back skin collected 24 h after the last Vaseline or IMQ application, and normalized with GAPDH (ST-Vaseline *n* = 25, ST-IMQ *n* = 32, LT-Vaseline *n* = 28, LT-IMQ *n* = 26). A Two-Way ANOVA with Bonferroni’s correction was performed to compare each mediator expression after ST and LT treatments either with Vaseline or IMQ.

Although we showed a trend to an increase of *Il17a* gene expression in the skin of ST-IMQ-treated mice compared to Vaseline, significative *Il17a* overexpression was only observed in LT-IMQ-treated mice (Fig. 2a). Identical overexpression of antimicrobial peptide beta-defensin 3 *(Bd3)*, interleukin-1α (*Il1α*) and keratin 6 *(Krt6)* genes was observed following both ST- and LT-IMQ treatments, without significant differences between ST and LT treatments (Fig. 2b, c,d). However, the neutrophil-attracting chemokine *Cxcl3* gene was only increased in the skin of ST-IMQ-treated mice (Fig. 2e). Concerning epidermal differentiation, overexpression of suprabasal differentiation markers like keratin 10 *(Krt10)*, involucrin (*Ivl*) and kallikrein 5 (*Klk5*) was only observed in LT-IMQ-treated mice (Fig. 2f, g, h).

### Short-term IMQ treatment induces sharper epidermal features compared to long-term treatment

**Fig. 3:**
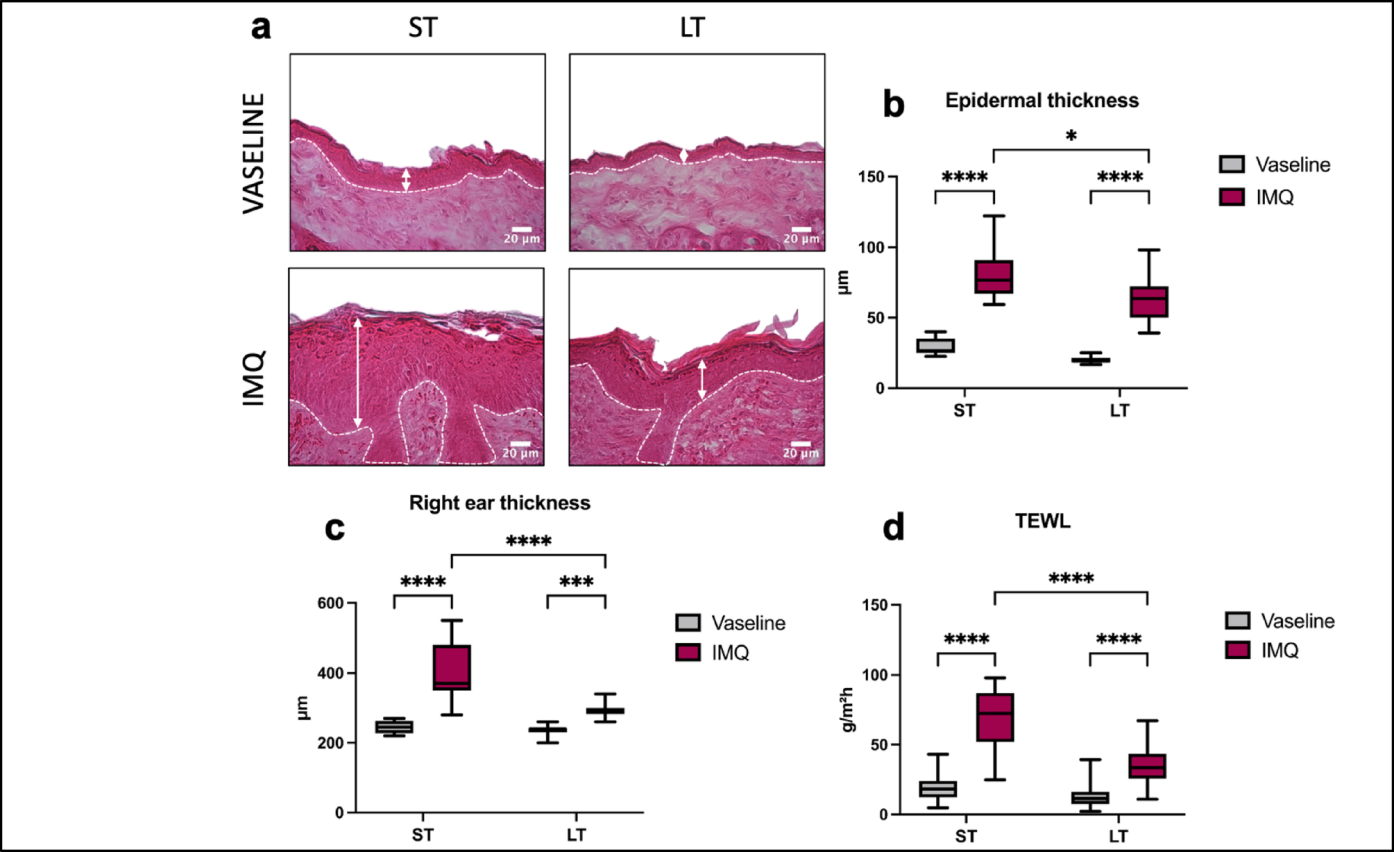
Effects of IMQ on the epidermal compartment after ST and LT treatments. (**A**) Representative images of back skin sections stained with hematoxylin and eosin after ST and LT treatments with Vaseline or IMQ, imaged with a x40 magnification. Scale bar: 20 μm. (**B**) Average epidermal thickness measured on back skin sections of ST- and LT-treated mice. The average thickness of an individual’s epidermis was measured over 4 to 6 fields imaged with a x10 magnification. (ST-Vaseline *n* = 10, ST-IMQ *n* = 10, LT-Vaseline *n* = 10, LT-IMQ *n* = 10). (**C**) Treated right ear thickness in ST- and LT-treated mice, performed using a micrometer (ST-Vaseline *n* = 14, ST-IMQ *n* = 15, LT-Vaseline *n* = 20, LT-IMQ *n* = 20). (**D**) Trans-epidermal Water Loss (TEWL) measurements were performed on two distinct areas of the treated back skin for each individual (ST-Vaseline *n* = 69, ST-IMQ *n* = 66, LT-Vaseline *n* = 63, LT-IMQ *n* = 62). Functional experiments and sample collection were performed 24 h following the last application of Vaseline or IMQ for both treatment durations. A Two-Way ANOVA with Bonferroni’s correction was performed to compare epidermis thickness, ear thickness and TEWL after ST and LT treatments either with Vaseline or IMQ.

Epidermis measurements performed on back skin histological sections confirmed that IMQ induced epidermal thickening after both ST- and LT-IMQ treatments compared to Vaseline (Fig. 3a, b). However, epidermal thickness was significantly greater after ST-IMQ treatment (81.2 ± 18.4 μm) compared to LT-IMQ treatment (63.5 ± 16.9 μm) (Fig. 3b). Total ear thickness measured *in vivo* using a micrometer confirmed greater thickening after ST-IMQ treatment compared to LT-IMQ treatment (Fig. 3c).

Regarding the functional integrity of epidermal barrier function, both ST and LT treatments with IMQ significantly increased trans-epidermal water loss (TEWL) compared to Vaseline (Fig. 3d). Interestingly, TEWL values were significantly higher after ST-IMQ treatment (70 ± 20 g/m²/h) compared to LT-IMQ treatment (35 ± 14 g/m²/h). Moreover, it is important to note that TEWL progressively returned closer to control values during untreated weeks of LT-IMQ treatment, suggesting rapid recovery following IMQ treatment cessation (Supplementary Fig. S3).

### Dermal features induced by IMQ do not differ according to treatment duration

**Fig. 4:**
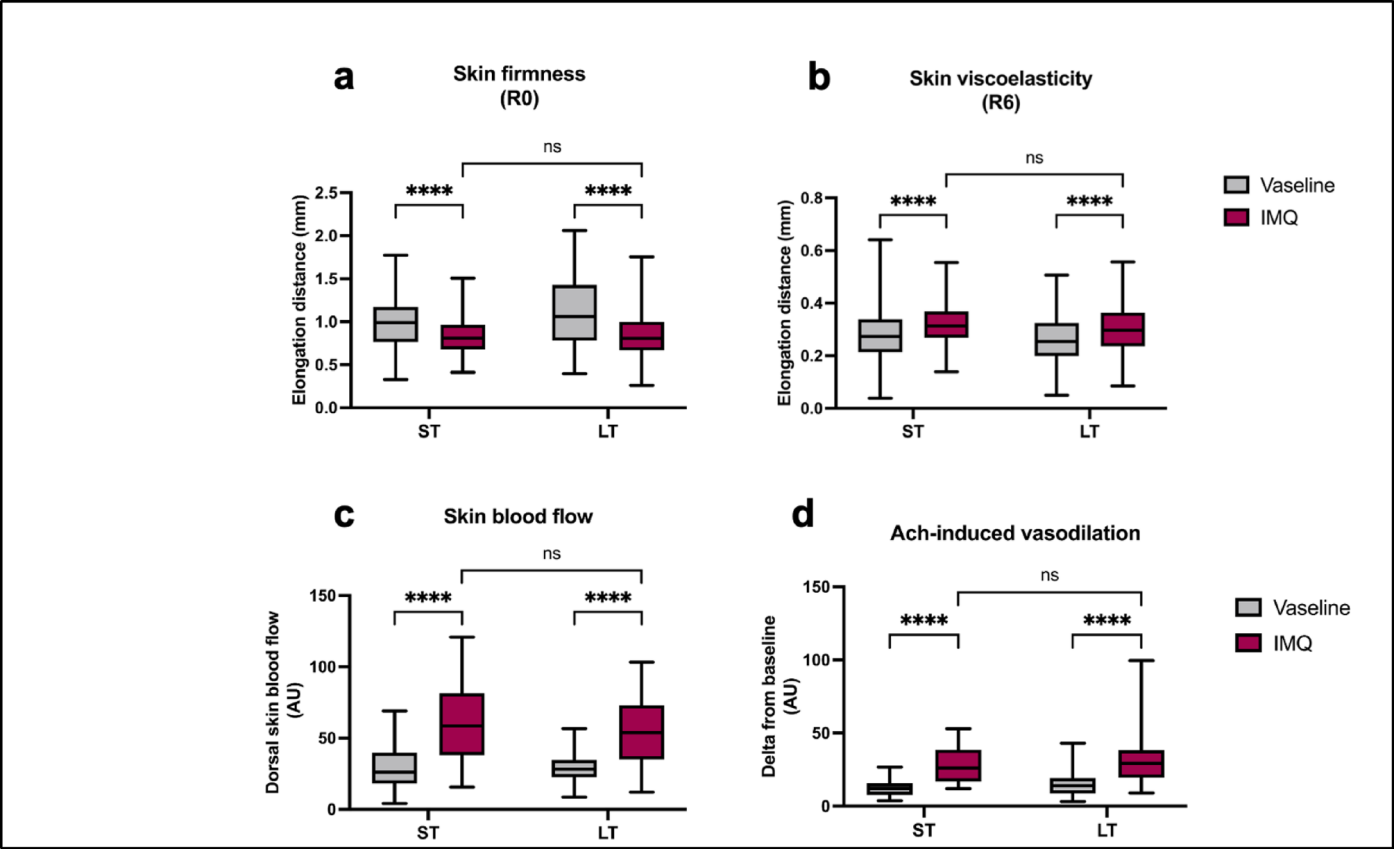
Effects of IMQ on the dermal compartment after ST and LT treatments. (**A**) Cutometer measurements of the R0 parameter reflecting skin firmness. A reduced R0 parameter indicates a firmer skin. (ST-Vaseline *n* = 69, ST-IMQ *n* = 63, LT-Vaseline *n* = 64, LT-IMQ *n* = 60). (**B**) Cutometer measurments of the R6 parameter reflecting skin viscoelasticity. An increased R6 parameter indicates a prevalence of the viscoelastic part over the elastic part of skin deformation. (ST-Vaseline *n* = 70, ST-IMQ *n* = 63, LT-Vaseline *n* = 65, LT-IMQ *n* = 60). (**C**) AUC of basal skin blood flow measured on the back skin of anaesthetized mice using doppler laser. After 1 min of baseline recording, Ach iontophoresis (**D**) was performed to assess the endothelium-dependent vasodilation (ST-Vaseline *n* = 37, ST-IMQ *n* = 24, LT-Vaseline *n* = 35, LT-IMQ *n* = 29). All functional experiments were performed 24 h following the last application of Vaseline or IMQ for both treatment duration. A Two-Way ANOVA with Bonferroni’s correction was performed to compare skin mechanical properties and endothelial reactivity after ST and LT treatments either with Vaseline or IMQ.

Concerning dermal mechanical properties, IMQ induced a significant and similar increase in skin firmness (characterized by a decrease of the R0 parameter) after ST (-16%) and LT (-20%) treatments compared to Vaseline (Fig. 4a). Similarly, skin elasticity was reduced by IMQ (characterized by an increase in the R6 parameter), with no difference between ST (+ 16%) and LT (+ 15%) treatments compared to Vaseline (Fig. 4b). Interestingly, skin firmness decreased during the untreated weeks of the LT-IMQ treatment, getting closer to control values of Vaseline-treated mice (Supplementary Fig.S3).

Next, we investigated the functionality of dermal blood vessels using Doppler laser. Here, IMQ increased basal skin blood flow in a similar manner after ST (+ 99.8%) and LT (+ 93.1%) treatments compared to Vaseline, demonstrating local inflammation as expected in psoriasis-like conditions (Fig. 4c). Then, we measured the endothelium-dependent vasodilation in response to iontophoretic delivery of acetylcholine (Ach). Ach-induced vasodilation was increased by IMQ after both ST (+ 126%) and LT (+ 115%) treatments compared to Vaseline (Fig. 4d). Thus, these results demonstrate an equivalent exacerbated vascular reactivity induced by IMQ, regardless of IMQ treatment duration.

### Treatment duration does not strikingly influence the correlation of cutaneous gene expression between IMQ-induced dermatitis and human psoriasis

**Fig. 5:**
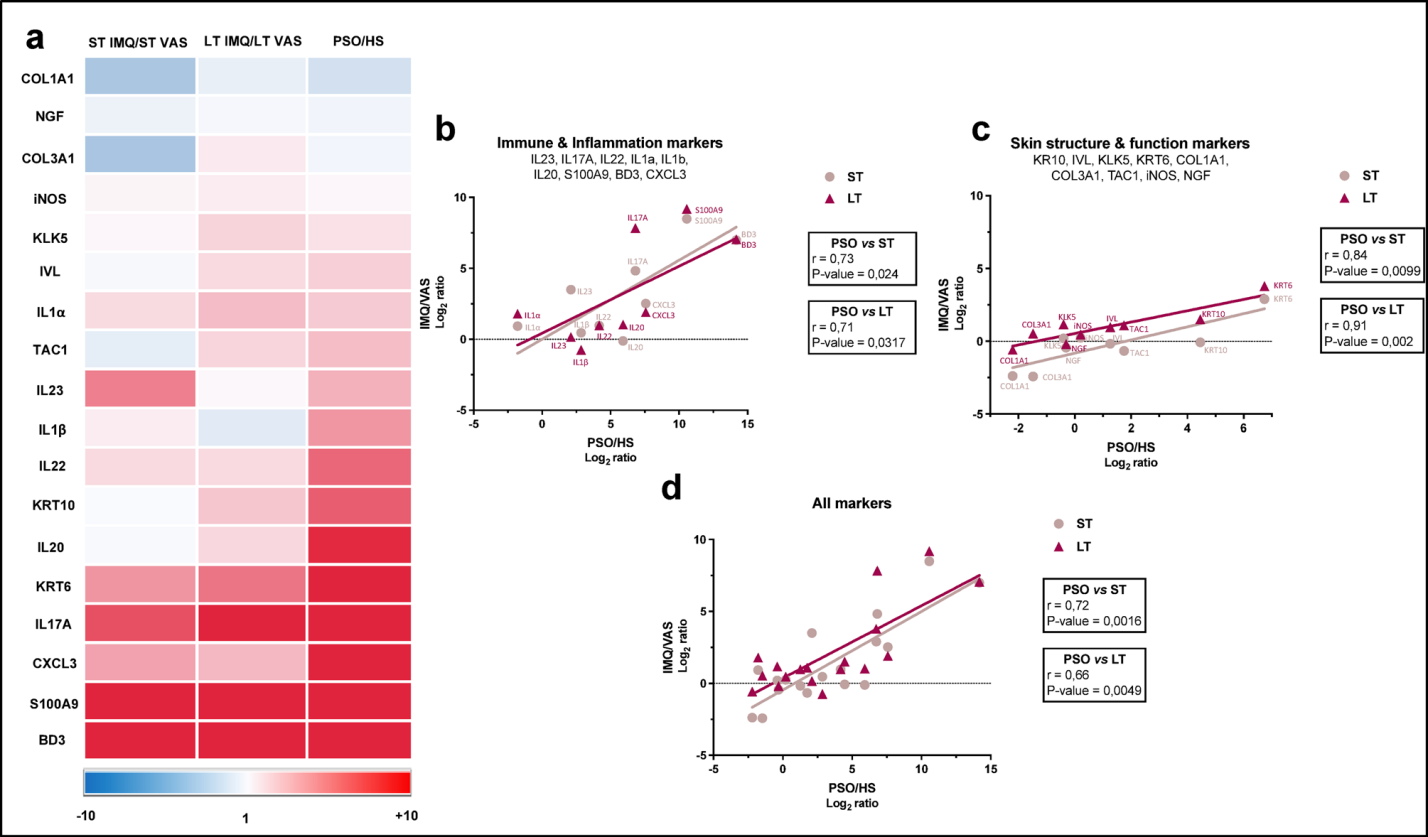
Comparison of cutaneous gene expression profile between ST-IMQ treatment, LT-IMQ treatment and human psoriasis. (**A**) Heat map representing the Log_2_ fold-change values of differentially expressed genes during ST-IMQ treatment (ST), LT-IMQ treatment (LT) compared to Vaseline treatment and in human lesional psoriatic skin (PSO) compared to human healthy skin (HS). The value of fold-change between ST, LT treatments and human psoriasis for each gene is indicated by the colored scale, with red indicating a value > 1 and blue indicating a value < 1. (ST-Vaseline *n* = 25, ST-IMQ *n* = 29, LT-Vaseline *n* = 27, LT-IMQ *n* = 28, PSO *n* = 65, HS *n* = 17). (**B**) Pearson correlation of Log_2_ ratios for genes encoding **(B)** immune and inflammation markers (IL23, IL17A, IL22, IL1α, IL1β, IL20, S100A9, BD3 and CXCL3) or (**C**) skin structure and function markers (KRT10, IVL, KLK5, KRT6, COL1A1, COL3A1, TAC1, iNOS and NGF). (**D**) Spearman correlation of Log_2_ ratios for genes encoding all of the markers present on the heat map, without distinction in gene subfamilies.

Here, we compared the expression profiles of some typical psoriasis markers in the lesional skin of ST-IMQ treated mice, LT-IMQ treated mice and human patients with psoriasis to evaluate the adequacy of acute and/or chronic IMQ treatments in mimicking human psoriasis. We focused on the expression of several psoriasis-related cytokines (*IL-17A*,* IL-20*,* IL-23*,* IL-1α*,* IL-1β*), chemokines (*CXCL3*) and AMPs (*S100A9*,* BD3*) but also on epidermal markers (*KRT10*,* KRT6*,* IVL*,* KLK5*), dermal markers (*COL1A1*,* COL3A1*) and molecules implicated in vasodilation (*NGF*,* iNOS*,* TAC1*), all known to be differentially expressed in human lesional psoriatic skin compared to healthy skin. The heatmap analysis highlighted similarities in the regulation of gene expression between ST-IMQ treatment, LT-IMQ treatment and human psoriasis (e.g. *S100A9*,* BD3 or IL-17 A*) (Fig. 5a). It also revealed some discrepancies between ST-IMQ and LT-IMQ treatments (e.g. *COL3A1*,* TAC1*, *IL-23*,* IL-20 or IL-1β*) and between mouse and human psoriatic skin (e.g. *IL-20 or IL-1β*). To have a clearer idea of the correlation strength between either ST- or LT-IMQ treatments and human psoriasis based on the genic regulation of key psoriasis markers, we performed correlation test on the Log2 fold-change values of differentially expressed genes in human psoriatic skin compared to healthy skin and in ST- and LT-IMQ-treated mice skin compared to Vaseline-treated skin (Fig. 5b, c, d). We observed a comparable positive correlation in the transcriptional regulation of immune and inflammation markers including cytokines, chemokines and AMPs between both IMQ treatments and human psoriasis (r_*PSO vs. ST*_ = 0.73; r_*PSO vs. LT*_ = 0.71) (Fig. 5b) but a stronger correlation for skin structure and function markers including epidermal, dermal and vascular markers between LT-IMQ and PSO than between ST-IMQ and PSO (r_*PSO vs. ST*_ = 0.84; r_*PSO vs. LT*_ = 0.91) (Fig. 5c). However, the correlation analysis of transcriptional regulation for all analysed genes without distinction in subfamilies also reported a positive correlation with a slightly higher correlation for PSO vs. ST-IMQ treatment than PSO vs. LT-IMQ treatment (r_*PSO vs. ST*_ = 0.72, p-value = 0.0016; r_PSO vs. LT_ = 0.66, p-value = 0.0033) (Fig. 5d).

## Discussion

The aim of this study was to evaluate the differences between short-term (ST) and long-term (LT) treatments with imiquimod (IMQ) on their ability to induce cutaneous psoriasis features at the macroscopic, histological, molecular and functional levels in mouse skin. We also evaluated the capacity of these two treatment durations to mimic human psoriasis based on the transcriptional regulation of psoriasis-related factors.

Our results showed that both ST- and LT-IMQ treatments were well tolerated by healthy young mice, despite transient weight loss. Both treatments induced psoriasiform dermatitis characterized by redness, skin thickening and desquamation, as previously described^13,18^. While the overall severity of lesions, assessed by a PASI-like score, was similar for both treatments, specific characteristics varied according to treatment duration. Since IMQ concentration in the skin was similar at the end of both treatments, these differences likely stem from the acute versus chronic nature of the dermatitis. In particular, redness was more accentuated after ST-IMQ treatment, indicating a more pronounced inflammation. Additionally, epidermal thickness was greater after ST-IMQ than LT-IMQ treatment when measured on back skin sections or on the ear of living mice, although no statistical difference was observed during clinical evaluation (thickness score). In accordance, desquamation was more significant after LT-IMQ treatment, in association with increased expression of involucrin and kallikrein 5, both linked to excessive desquamation^19^ and both up-regulated in patients with chronic psoriasis^20^. Among the few studies that have investigated cutaneous features induced by extended IMQ treatments, Vasseur et al., reported that LT-IMQ treatment induced sharper erythema and epidermal hyperplasia compared to ST treatment. However, another study comparing 6-days and 12-days long IMQ treatments found no significant differences in epidermal thickness between treatment durations^21^. These differences could be explained by the different methods used to measure epidermal hyperplasia. In our study, we demonstrated reduced skin thickening following LT-IMQ treatment by using both an unbiased approach integrating the total area of the epidermis on histological sections as well as *in vivo* measurements of total ear thickness using a micrometer. These consistent findings, obtained through two separate methodologies across different anatomical locations, strengthen the conclusion that extended IMQ exposure does not inherently exacerbate skin thickening.

Beyond clinical signs, IMQ treatments also resulted in functional alterations at both epidermal and dermal levels. Trans-epidermal water loss (TEWL) was increased in lesional skin after both ST- and LT-IMQ treatments, indicating compromised skin barrier function, as previously reported in human psoriasis and ST-IMQ mouse model^2,22^. Notably, the present study provides the first in vivo demonstration of impaired epidermal barrier function in LT-IMQ treatment, which appeared less severe than in ST-IMQ treatment. Regarding dermal mechanical properties, both skin firmness and elasticity were affected by IMQ, regardless of treatment duration, as previously reported in psoriasis patients^4^. Changes in these properties likely stem from modifications in the expression of extracellular matrix regulators such as MMP9, HPSE or SYND^23,24^. Moreover, as expected in inflamed skin^25^basal skin blood flow was increased after ST and LT-IMQ treatments. This increase aligns with findings in the lesional skin of psoriasis patients and ST-IMQ mouse models^8,26^. Cutaneous perfusion measured in human healthy skin exposed to short (2–3 days) and long (5–7 days) IMQ applications was not different between the two durations of treatment^27 ^strengthening the observation that IMQ treatment duration does not necessarily influence cutaneous perfusion. Moreover, endothelial dysfunction, which is known to be driven by an imbalance in vasoconstrictor and vasodilator factors^7^was also observed after both treatments, evidenced by an increased response to acetylcholine (Ach) iontophoresis in lesional skin. This exacerbated cutaneous Ach-induced vasodilation parallels findings in low-grade inflammation contexts, such as in obese mice^28^ or LPS-induced inflammation^29^. However, inverse effects on other vascular territories, such as more distal vessels or those of larger caliber cannot be excluded^30^.

Our second objective was to evaluate the ability of both ST and LT-IMQ treatments to mimic human psoriasis by comparing the expression profile of typical psoriasis markers in the skin of ST-IMQ treated mice, LT-IMQ treated mice and human lesional psoriatic areas. While some studies have compared ST-IMQ treatment with human lesional psoriasis^31,32^no prior comparisons of gene expression between LT-IMQ treatment in mice and human lesional psoriatic skin have been made. Our findings demonstrated similar transcriptional regulation of cytokines, chemokines, antimicrobial peptides (AMPs) across ST-IMQ, LT-IMQ and human psoriatic skin, with no significant differences based on treatment duration. However, transcriptional regulation of epidermal, dermal and vascular markers showed a better correlation between LT-IMQ treatment and psoriasis than between ST-IMQ treatment and psoriasis. For example, genes encoding epidermal differentiation proteins such as *IVL*, *KRT10* or *KLK5*, all known to be dysregulated in patients suffering from psoriasis^20,33,34^were overexpressed in the skin of LT-IMQ-treated mice compared to ST-IMQ treated mice. In addition, the Log2 ratios of *IVL*, *KRT10* or *KLK5* expression after LT-IMQ treatment were all closer to the corresponding Log2 ratios of human patients with psoriasis, suggesting that disruption of epidermal differentiation is a hallmark of chronic inflammation, which is elegantly translated in the LT-IMQ treatment model. However, analysis of all markers, without distinction in subfamilies, revealed that the regulation of psoriasis-related genes in ST-IMQ treatment aligned more closely with human lesional psoriatic skin than in LT-IMQ treatment, although the difference between the two treatments remained subtle. Because several studies demonstrated that ST-IMQ treatment induces less T cell recruitment compared to human psoriasis^32^likely due to species differences in TLR7 expression or unidentified TLR7-independent mechanisms leading to psoriasiform dermatitis^35^its ability to truly reflect human psoriasis is often questioned. It therefore seems interesting to further conduct a detailed characterization of immune cell recruitment in the skin of both ST-IMQ and LT-IMQ treated mice to determine if LT-IMQ treatment could induce increased abundance of T cells and therefore more accurately mimics human psoriasis.

Finally, we demonstrated that both cumulative clinical score and skin functionality measurements (TEWL and cutometer), which were all altered during IMQ-treated weeks of the LT protocol, returned to control levels during untreated weeks. This pattern suggests a global restoration of skin integrity within 9 days following IMQ treatment cessation, consistent with previous study showing skin recovery within 10 days^21^. Furthermore, a study comparing short (2–3 days) and long (5–7 days) IMQ exposures in human healthy skin previously showed that erythema and increased skin perfusion recovered within 7 days after discontinuation of IMQ treatment^27^. If these findings imply that an optimal model for IMQ-induced chronic inflammation would require continuous IMQ application to prevent intermittent skin recovery on untreated periods, uninterrupted exposure over 12 days leads to severe dehydration in mice, prompting the adoption of a nine-weeks-long protocol with alternating weeks of treatment. A notable limitation of the LT-IMQ treatment was hair regrowth, which required shearing of the mice and additional isoflurane anaesthesia compared to the ST-IMQ treatment. Despite these constraints and the observed recovery of skin parameters during untreated intervals, we identified significant differences between ST-IMQ and LT-IMQ treatments. Although intermittent recovery occurred during the LT protocol, underlying inflammation appears to persist, likely at a reduced level or via alternative mechanistic pathways, which could explain the differences at the end of ST and LT treatments. In support of this hypothesis, a six-day-long exposure to IMQ in mice induced overexpression of IL-23 and IL-17, which returned to basal levels by day 12 of IMQ treatment while clinical signs of the disease persisted, indicating a sustained yet potentially compensated inflammatory loop^21^. Interestingly, prolonged IMQ exposures in human healthy (5–7 days) skin activate additional molecular pathways, such as IRF-7 signaling, but also additional recruitment of T cells compared to shorter exposures (2–3 days), strengthening the fact that prolonged exposure to IMQ implies several distinct molecular pathways compared to shorter exposures^27^. Moreover, the capacity of TLR7, which binds IMQ, to be desensitized over repetitive stimulations, could also be a possible explanation for reduced erythema, epidermal hyperplasia and skin barrier function disruption in LT treatment compared to ST treatment^36^.

In conclusion, this study showed that both ST and LT-IMQ treatments can induce psoriasis-like cutaneous features at the clinical, histological, molecular and functional levels. Treatment duration notably influenced epidermal features, with ST treatment causing more pronounced epidermal hyperplasia and epidermal barrier disruption than LT treatment. However, IMQ treatment duration did not significantly affect dermal features, including skin stiffening and endothelial dysfunction. These slight but important differences raised new points to improve the refinement of the IMQ model and provide valuable insights for selecting the appropriate IMQ treatment duration based on specific research objectives.

## Materials and methods

### Human skin biopsies

This investigation is an ancillary study of the IMPRA (NCT03389984) study in which skin inflammatory transcriptomic profiles have been studied in psoriatic patients, before and after adalimumab therapy. This study was conducted according to the Declaration of Helsinki principles and oral informed consent was obtained from participants before inclusion. Eligible patients had chronic plaque psoriasis with a Psoriasis Area and Severity Index (PASI) ranging from 10 to 50 (mean = 17.1) and body surface area (BSA) involvement ≥ 10%, without anti-psoriatic biotherapy for six months, other systemic treatment for four weeks, or topical therapy for two weeks preceding enrolment. Skin biopsies were collected from lesional skin of patients with psoriasis at diagnosis (*n* = 65) and immediately frozen in liquid nitrogen before further RNA extraction. Healthy skin biopsies (*n* = 17) were obtained from surgical samples of brachioplasty, healthy breast or abdominal skins provided by the Plastic Surgery Unit of Poitiers Hospital (DC2014-2109). The use of human skin samples for research studies was approved by the Ethical Committee of Poitiers Hospital.

### Animals

Eight-week-old male and female C57BL6/J mice were purchased from Janvier Labs (Le Genest-Saint-Isle, France). Before the beginning of experiments, a stabling period of 7 days allowed the mice to get used to their environment consisting in ad libitum access to food and water, a temperature-controlled room (22–23 °C) with a 12-hour light/dark cycle. The experiments of this project were approved by scientific and ethics committees (CECCAPP, Lyon, France) under the code LBTI-2020-007 and registered under Apafis number #28,285, approved on 27 November 2020. All experiments were conducted in accordance with European Union recommendations for animal experimentation. This study was carried out in compliance with the ARRIVE guidelines (www.arriveguidelines.org).

### Psoriasiform dermatitis induction with short-term (ST) and long-term (LT) imiquimod treatments

The back skin of mice was shaved and depilated under anaesthesia with isoflurane at least 48 h before the beginning of IMQ or Vaseline treatments. In IMQ groups, Aldara cream containing 5% of IMQ (Viatris, Ireland) was applied on the lower back skin (62.5 mg) and the right ear (13 mg), whereas Vaseline was applied to serve as controls. Topical treatment (IMQ or Vaseline) was applied during 4 consecutive days for ST groups and for 9 weeks (one week out of two) for LT groups (5 consecutive days per week, except for the 9th week) (Supplementary Fig. S4). Mice were divided into the following groups: ST-Vaseline (*n* = 82), ST-IMQ (*n* = 77), LT-Vaseline (*n* = 66), LT-IMQ (*n* = 64), with an equivalent number of males and females. The exact numbers of mice for each studied parameter are mentioned in the figure legends. All IMQ-treated mice were injected intraperitoneally with 0.9% NaCl solution each day of treatment to prevent dehydration. All experiments and biological samples collection were performed 24 h after the last cream application. No additional depilation was performed during the 9 weeks of treatment to prevent the effects of depilatory cream from interfering with or biasing our results. When necessary (no more than 10 times over the total duration for both treatments), hair removal was performed with a shearing method in anaesthetized mice (isoflurane).

### General evaluation of mouse skin inflammation

The severity and extent of the IMQ-induced cutaneous inflammation was visually measured on the back skin of mice using an objective clinical scoring method based on the clinical Psoriasis Area Severity Index (PASI), as previously described^37^. In addition, treated-ear thickness was measured at the end of ST and LT treatments using a micrometer (Türlen Anytime Tools, Granada Hills, CA, USA).

### Functional analyses on mouse skin

All functional analyses were performed on the depilated back skin of anaesthetized mice (intraperitoneal injection of thiopental, 75mg.kg^− 1^). Transepidermal water loss (TEWL) was assessed using the Aquaflux equipment (Biox Systems Ltd., London, UK). Skin firmness (R0 parameter) and visco-elasticity (R6 parameter), reflecting dermal mechanical properties, were measured using the 2 mm probe of the Cutometer MPA 580 (Courage and Khazaka, Köln, Germany). Skin blood flow was measured using a laser Doppler probe (481-1, Perimed Sweden). The endothelium-dependent responses were assessed following iontophoretic delivery of acetylcholine (Ach) (Ach 2%, using an anodal current application of 100 mA for 20 s). Vasodilation was expressed as the maximum increase in blood flow obtained over the entire recording period (20 min) following iontophoretic delivery from baseline (calculated prior to iontophoresis), as previously described^38^.

### Gene expression in human and mouse skin

Total RNA was isolated from human and mouse skin biopsies. RNA was extracted using a NucleoSpin RNA II kit (Macherey-Nagel, Hoerdt, France) and reverse-transcribed with the SuperScript II reverse transcriptase (Invitrogen) according to the manufacturer’s instructions. Quantitative PCR was conducted using the AceQ Universal SYBR qPCR Master Mix kit (Vazyme, Clinisciences, Nanterre, France) and a LightCycler 480 system (Roche Diagnostics, Meylan, France). Relative RNA expression was determined according to the ΔCT method (relative expression = 2 exp(ΔCT) = 2 exp(CT target – CT glyceraldehyde-3-phosphate dehydrogenase (GAPDH)). All primer sequences used in this study are available in Supplementary Table S1.

### Histological analysis of mouse skin

Mouse back skin samples were immediately fixed in 4% paraformaldehyde overnight and cryopreserved in a 10% sucrose solution. Samples were frozen in isopentane cooled by liquid nitrogen and stored at -80 °C until further analysis. Sections of 10 μm thickness underwent haematoxylin and eosin (H&E) staining. For evaluation of epidermal thickness, 4 to 6 fields by individual were pictured, at a X10 magnification, under a light microscope (Axiostar plus, Carl Zeiss, Oberkochen, Germany). For each field, the epidermis area was divided by the length of the epidermis to obtain the average thickness of the epidermis in µm.

### Statistical analysis

Two-way analysis of variance followed by Bonferroni’s multiple comparison tests were used to determine significance between the duration (ST vs. LT) and type (Vaseline vs. IMQ) of treatments. Spearman and Pearson rank correlation analysis was performed to assess the correlation of gene transcriptional regulation between ST or LT-IMQ mouse skin and human lesional psoriatic skin. All tests were performed using PRISM software (GraphPad Software version 10.0, San Diego, CA, USA). Results are expressed as mean ± SD and mean differences were considered statistically significant when *p* < 0.05. *p < 0.05, **p < 0.01, ***p < 0.001, ****p < 0.0001.

## Electronic supplementary material

Below is the link to the electronic supplementary material.

Supplementary Material 1

## Data Availability

The datasets generated during and/or analysed during the current study are available from the corresponding author on reasonable request.

## References

[1] World Health Organization. *Global Report on Psoriasis* (World Health Organization, 2016).

[2] Nikam, V., Monteiro, R., Dandakeri, S. & Bhat, R. Transepidermal water loss in psoriasis: A case-control study. *Indian Dermatol. Online J.* **10**, 267 (2019). 31149569 10.4103/idoj.IDOJ_180_18 PMC6536057

[3] Morariu, S. H. et al. Epidermal barrier parameters in psoriasis: implications in assessing disease severity. *J. Pers. Med.* **14**, 728 (2024). 39063982 10.3390/jpm14070728 PMC11278309

[4] Dobrev, H. In vivo study of skin mechanical properties in psoriasis vulgaris. *Acta Derm Venereol.* **80**, 263–266 (2000). 11028858 10.1080/000155500750012135

[5] Bu, J., Ding, R., Zhou, L., Chen, X. & Shen, E. Epidemiology of psoriasis and comorbid diseases: A narrative review. *Front. Immunol.* **13**, 880201 (2022). 35757712 10.3389/fimmu.2022.880201 PMC9226890

[6] Abuabara, K. et al. Cause-specific mortality in patients with severe psoriasis: a population-based cohort study in the U.K.: Cause-specific mortality in patients with severe psoriasis. *Br. J. Dermatol.* **163**, 586–592 (2010). 20633008 10.1111/j.1365-2133.2010.09941.x PMC2966545

[7] Alba, B. K., Greaney, J. L., Ferguson, S. B. & Alexander, L. M. Endothelial function is impaired in the cutaneous microcirculation of adults with psoriasis through reductions in nitric oxide-dependent vasodilation. *Am. J. Physiol. -Heart Circ. Physiol.* **314**, H343–H349 (2018). 29054972 10.1152/ajpheart.00446.2017 PMC5867651

[8] Yosipovitch, G., Chan, Y. H., Tay, Y. K. & Goh, C. L. Thermosensory abnormalities and blood flow dysfunction in psoriatic skin. *Br. J. Dermatol.* **149**, 492–497 (2003). 14510980 10.1046/j.1365-2133.2003.05585.x

[9] Mahil, S. K. et al. Comparing the efficacy and tolerability of biologic therapies in psoriasis: an updated network meta-analysis. *Br. J. Dermatol.* **183**, 638–649 (2020). 32562551 10.1111/bjd.19325

[10] Brown, W. R. & Hardy, M. H. A hypothesis on the cause of chronic epidermal hyperproliferation in Asebia mice. *Clin. Exp. Dermatol.* **13**, 74–77 (1988). 3214959 10.1111/j.1365-2230.1988.tb00661.x

[11] Sundberg, J. P. et al. Development and Progression of Psoriasiform Dermatitis and Systemic Lesions in the Flaky Skin (*fsn*) Mouse Mutant. *Pathobiology* 65, 271–286 (1997). 10.1159/000164138 9459497

[12] Wrone-Smith, T. & Nickoloff, B. J. Dermal injection of immunocytes induces psoriasis. *J. Clin. Invest.* **98**, 1878–1887 (1996). 8878440 10.1172/JCI118989 PMC507628

[13] van der Fits, L. et al. Imiquimod-Induced Psoriasis-Like skin inflammation in mice is mediated via the IL-23/IL-17 Axis. *J. Immunol.* **182**, 5836–5845 (2009). 19380832 10.4049/jimmunol.0802999

[14] Jabeen, M. et al. Advanced characterization of Imiquimod-Induced Psoriasis-Like mouse model. *Pharmaceutics* **12**, E789 (2020). 10.3390/pharmaceutics12090789 PMC7558091 32825447

[15] Xia, Y. P. et al. Transgenic delivery of VEGF to mouse skin leads to an inflammatory condition resembling human psoriasis. *Blood* **102**, 161–168 (2003). 12649136 10.1182/blood-2002-12-3793

[16] Sano, S. et al. Stat3 links activated keratinocytes and immunocytes required for development of psoriasis in a novel Transgenic mouse model. *Nat. Med.* **11**, 43–49 (2005). 15592573 10.1038/nm1162

[17] Gangwar, R. S., Gudjonsson, J. E. & Ward, N. L. Mouse models of psoriasis: A comprehensive review. *J. Invest. Dermatol.* **142**, 884–897 (2022). 34953514 10.1016/j.jid.2021.06.019 PMC10190156

[18] Vasseur, P. et al. Liver fibrosis is associated with cutaneous inflammation in the imiquimod-induced murine model of psoriasiform dermatitis. *Br. J. Dermatol.* **179**, 101–109 (2018). 29150843 10.1111/bjd.16137

[19] Furio, L. et al. Transgenic Kallikrein 5 mice reproduce major cutaneous and systemic hallmarks of Netherton syndrome. *J. Exp. Med.* **211**, 499–513 (2014). 24534191 10.1084/jem.20131797 PMC3949577

[20] Zingkou, E., Pampalakis, G. & Sotiropoulou, G. Keratinocyte differentiation and proteolytic pathways in skin (patho) physiology. *Int. J. Dev. Biol.* **66**, 269–275 (2022). 34881788 10.1387/ijdb.210161gs

[21] Tran, P. T. T., Le, L. T., Le, T. T. V., Van, T. T. & Vu, N. B. Effects of imiquimod application durations on Psoriasis-like lesions and cytokine expression in mice. *Biomed. Res. Ther.* **11**, 6387–6401 (2024).

[22] Chuang, S. Y. et al. 2,4-Dimethoxy-6-Methylbenzene-1,3-diol, a benzenoid from antrodia cinnamomea, mitigates psoriasiform inflammation by suppressing MAPK/NF-κB phosphorylation and GDAP1L1/Drp1 translocation. *Front. Immunol.* **12**, 664425 (2021). 34054833 10.3389/fimmu.2021.664425 PMC8162112

[23] Ma, F. et al. Single cell and Spatial sequencing define processes by which keratinocytes and fibroblasts amplify inflammatory responses in psoriasis. *Nat. Commun.* **14**, 3455 (2023). 37308489 10.1038/s41467-023-39020-4 PMC10261041

[24] Wagner, M. F. M. G., Theodoro, T. R., Filho, C. D. A. S. M., Oyafuso, L. K. M. & Pinhal, M. A. S. Extracellular matrix alterations in the skin of patients affected by psoriasis. *BMC Mol. Cell. Biol.* **22**, 55 (2021). 34715781 10.1186/s12860-021-00395-1 PMC8555298

[25] Huggenberger, R. & Detmar, M. The cutaneous vascular system in chronic skin inflammation. *J. Investig Dermatol. Symp. Proc.* **15**, 24–32 (2011). 22076324 10.1038/jidsymp.2011.5 PMC3398151

[26] Zhang, Y. et al. Factor XII and prekallikrein promote microvascular inflammation and psoriasis in mice. *Br. J. Pharmacol.* **181**, 3760–3778 (2024). 38872396 10.1111/bph.16428

[27] Van Den Noort, J. A. et al. Extending the IMQ Model: Deep Characterization of the Human TLR7 Response for Early Drug Development. *Inflammation* (2024). 10.1007/s10753-024-02127-x 10.1007/s10753-024-02127-x PMC12234578 39183259

[28] Nguyen-Tu, M. S. et al. Inflammation-linked adaptations in dermal microvascular reactivity accompany the development of obesity and type 2 diabetes. *Int. J. Obes.* **43**, 556–566 (2019). 10.1038/s41366-018-0148-4 PMC6223541 30006585

[29] Sanchez, B. et al. Skin Cell and Tissue Responses to Cross-Linked Hyaluronic Acid in Low-Grade Inflammatory Conditions. *Int. J. Inflamm.* 1–14 (2023). (2023). 10.1155/2023/3001080 PMC10474960 37663889

[30] Karbach, S. et al. Interleukin 17 drives vascular inflammation, endothelial dysfunction, and arterial hypertension in Psoriasis-Like skin disease. *Arterioscler. Thromb. Vasc Biol.* **34**, 2658–2668 (2014). 25341795 10.1161/ATVBAHA.114.304108

[31] Swindell, W. R. et al. Genome-Wide expression profiling of five mouse models identifies similarities and differences with human psoriasis. *PLoS ONE*. **6**, e18266 (2011). 21483750 10.1371/journal.pone.0018266 PMC3070727

[32] Swindell, W. R. et al. Imiquimod has strain-dependent effects in mice and does not uniquely model human psoriasis. *Genome Med.* **9**, 24 (2017). 28279190 10.1186/s13073-017-0415-3 PMC5345243

[33] Caputo, V. et al. Overview of the molecular determinants contributing to the expression of psoriasis and psoriatic arthritis phenotypes. *J. Cell. Mol. Med.* **24**, 13554–13563 (2020). 33128843 10.1111/jcmm.15742 PMC7754002

[34] Chen, J. Q. et al. Regulation of involucrin in psoriatic epidermal keratinocytes: the roles of ERK1/2 and GSK-3β. *Cell. Biochem. Biophys.* **66**, 523–528 (2013). 23283814 10.1007/s12013-012-9499-y

[35] Walter, A. et al. Aldara activates TLR7-independent immune defence. *Nat. Commun.* **4**, 1560 (2013). 23463003 10.1038/ncomms2566

[36] Michaelis, K. A. et al. Persistent Toll-like receptor 7 stimulation induces behavioral and molecular innate immune tolerance. *Brain Behav. Immun.* **82**, 338–353 (2019). 31499172 10.1016/j.bbi.2019.09.004 PMC6956569

[37] Moos, S., Mohebiany, A. N., Waisman, A. & Kurschus, F. C. Imiquimod-Induced psoriasis in mice depends on the IL-17 signaling of keratinocytes. *J. Invest. Dermatol.* **139**, 1110–1117 (2019). 30684554 10.1016/j.jid.2019.01.006

[38] Fromy, B. et al. Disruption of TRPV3 impairs Heat-Evoked vasodilation and thermoregulation: A critical role of CGRP. *J. Invest. Dermatol.* **138**, 688–696 (2018). 29054601 10.1016/j.jid.2017.10.006

