# Correction: Comparative analysis of cutaneous features of psoriasis in acute and chronic imiquimod-induced mouse models

**DOI:** 10.1038/s41598-025-25322-8

**Published:** 2025-10-28

**Authors:** Emma Fraillon, Jean-François Jégou, Hanitriniaina Rabeony, Jean-Claude Lecron, Nicolas Lebonvallet, Emilie Marie-Joseph, Audrey Josset-Lamaugarny, Géraldine Aimond, Laurent Misery, Franck Morel, Fabien P. Chevalier, Bérengère Fromy

**Affiliations:** 1 Laboratoire de Biologie Tissulaire et Ingénierie Thérapeutique, CNRS UMR5305, Université Claude Bernard Lyon 1, Lyon, France; 2 Laboratoire Inflammation, Tissus Épithéliaux et Cytokines, Université de Poitiers UR15560, Poitiers, France; 3 Centre Hospitalier et Universitaire (CHU) de Poitiers, Poitiers, France; 4 Laboratoire Interactions Épithéliums Neurones, Univ Brest UR4685, Brest, France; 5 Centre Hospitalier et Universitaire (CHU) de Brest, Brest, France

Correction to: *Scientific Reports* 10.1038/s41598-025-12111-6, published online 23 July 2025

The original version of this Article contained an error in Fig. 2, where statistics were missing. The original Fig. 2 and accompanying legend appear below

**Fig. 2:**
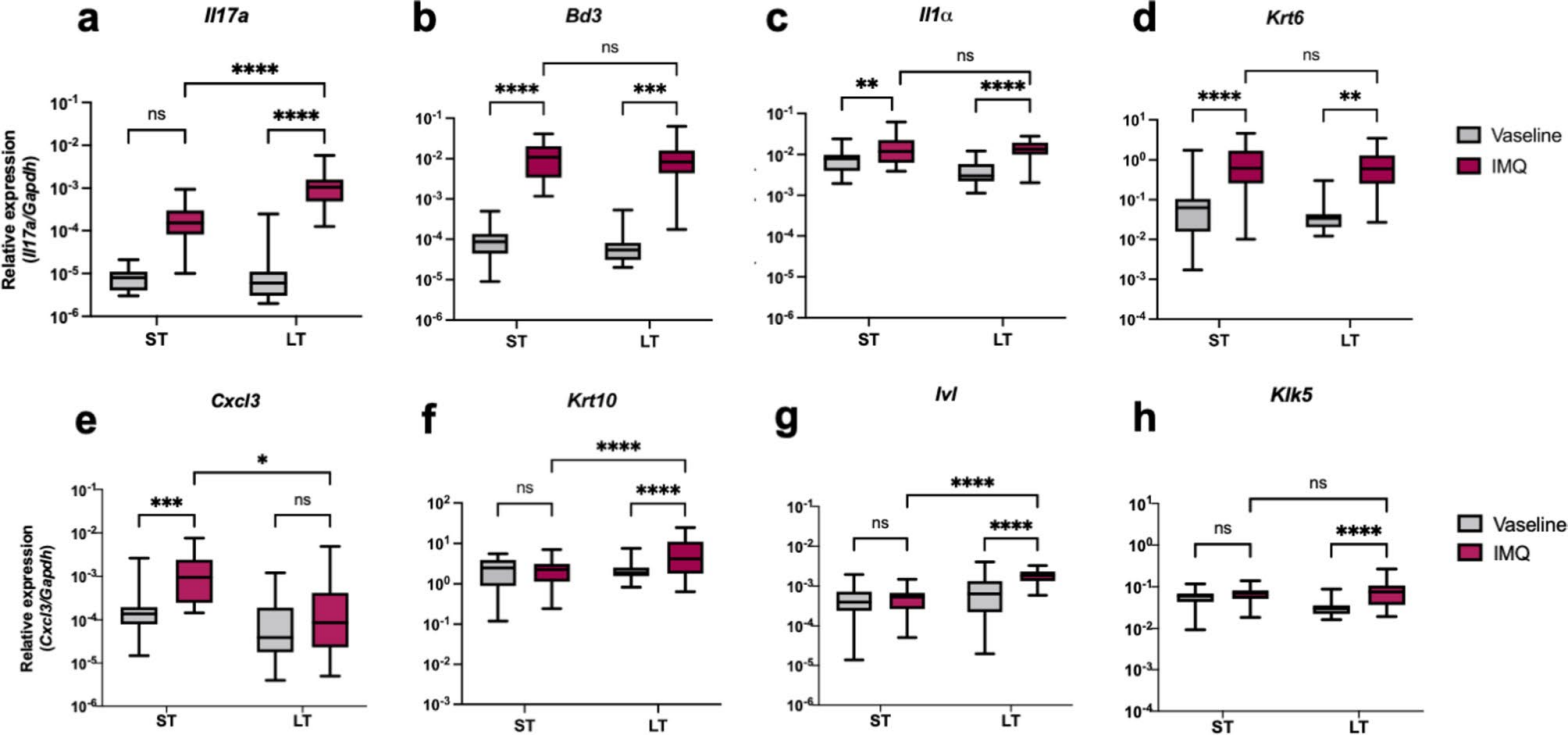
Effects of IMQ on the gene expression levels of pro-inflammatory mediators and epidermal markers after ST and LT treatments. Pro-inflammatory mediators (IL-17A (**A**), IL-1α (**B**), CXCL3 (**C**)), AMPs (BD3 (**D**)) and epidermal markers (KRT6 (**E**), KRT10 (**F**), IVL (**G**), KLK5 (**H**)) gene expression was assessed by RT-qPCR on total mRNAs from mouse back skin collected 24 h after the last Vaseline or IMQ application, and normalized with GAPDH (ST-Vaseline *n* = 25, ST-IMQ *n* = 32, LT-Vaseline *n* = 28, LT-IMQ *n* = 26). A Two-Way ANOVA with Bonferroni’s correction was performed to compare each mediator expression after ST and LT treatments either with Vaseline or IMQ.

The original Article has been corrected.

