# Supplementary material for: Comparative analysis of cutaneous features of psoriasis in acute and chronic imiquimod-induced mouse models
Source: Sci Rep. 2025 Jul 23;15:26834. doi: 10.1038/s41598-025-12111-6 (PMC12287311; doi:10.1038/s41598-025-12111-6)
Supplement: Supplementary file 1 — Supplementary Material 1 [file 41598_2025_12111_MOESM1_ESM.docx]

**Supplementary Figures**

**Supplementary Figure S1: Cumulative clinical score monitoring during LT treatment.**

Longitudinal monitoring of cumulative clinical score was assessed at the end of each treated week (1,3,5,7,9, grey) and just before restarting treatment (untreated weeks 2,4,6,8, white) of LT-Vaseline and LT-IMQ treatments.


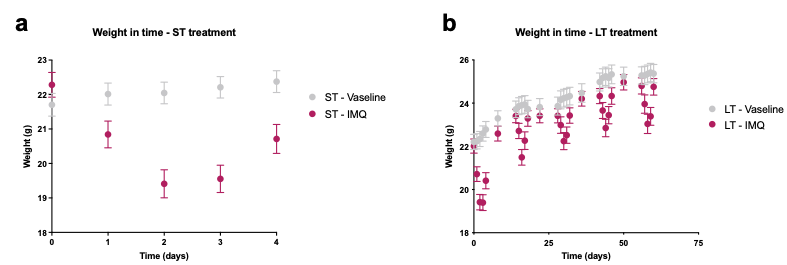


**Supplementary Figure S2: Body weight monitoring during ST and LT treatments.**

Longitudinal monitoring of body weight was performed daily for Vaseline and IMQ ST-treatments **(A)** and daily during treated (1,3,5,7,9) and once during untreated (2,4,6,8) weeks of Vaseline and IMQ LT-treatments **(B)**.

**Supplementary Figure S3: Longitudinal monitoring of epidermal barrier function and dermal mechanical properties during LT treatment.**

Longitudinal monitoring of **(A)** TEWL and **(B)** skin firmness (R0 parameter) on the back skin of mice at the end of the treated (1,3,5,7,9, grey) and untreated (2,4,6,8, white) weeks of LT-Vaseline and LT-IMQ treatments.


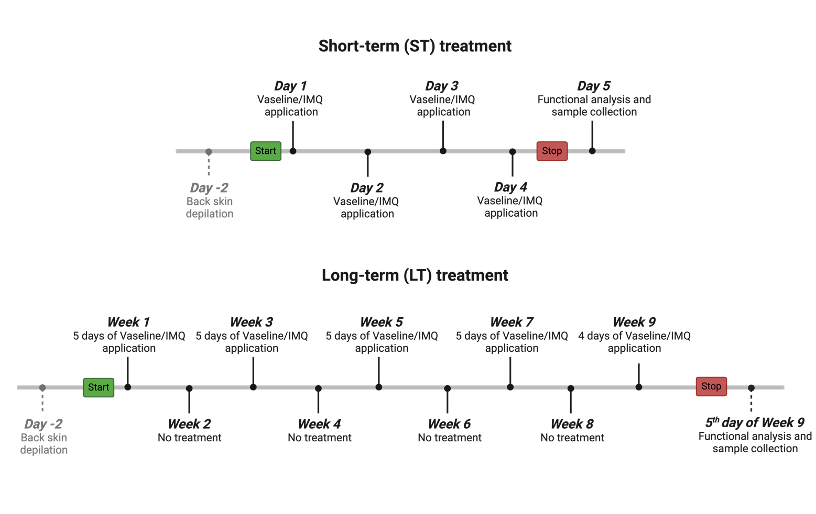


**Supplementary Figure S4: Detailed protocol for ST and LT treatments**.

For the ST treatment, Vaseline or IMQ was applied during four consecutive days on the depilated back skin and the right ear skin of mice. All functional analysis and sample collection were done on the fifth day, 24 hours after the last cream application. For the LT treatment, mice were treated with Vaseline or IMQ on the same anatomical areas and with similar cream quantities as ST treatment during nine weeks, every second weeks (five days of treatment followed by nine untreated days, during nine weeks). On the last week of LT treatment, mice were treated only four days and all functional analysis and sample collection were performed on the fifth day. This illustration was generated online using Biorender (https://www.biorender.com/).

**Supplementary Material**

| **Gene** | **Species** | **Forward sequence** | **Reverse sequence** |
| --- | --- | --- | --- |
| Col1a1 | Human | 5’- CGATGGATTCCAGTTCGAGTA -3’ | 5’- GTTTACAGGAAGCAGACAGG -3’ |
|  | Mouse | 5’- GCTACGCTGTTCTTGCAGTG -3’ | 5’- GCTACGCTGTTCTTGCAGTG -3’ |
| NGF | Human | 5’- ATACAGGCGGAACCACACTC -3’ | 5’- TGCTCCTGTGAGTCCTGTTG -3’ |
|  | Mouse | 5’- CCCAAGCTCACCTCAGTGTC -3’ | 5’- TGTACGGTTCTGCCTGTACG -3’ |
| Col3a1 | Human | 5’- GAAATGATGGTGCTCCTGGT -3’ | 5’- GTTCCCCAGGTTTTCCATTT -3’ |
|  | Mouse | 5’- GTCCAGGGATACGGGGTATG -3’ | 5’- CAGGGAAACCCATGACACCA -3’ |
| iNOS | Human | 5’- AGTCCCGAAGTTCTCAAGGC-3’ | 5’- AGGGGGCTTTCTCCACATTG -3’ |
|  | Mouse | 5’- CAGAGGACCCAGAGACAAGC -3’ | 5’- CATCCAGAGTGAGCTCCTAGG -3’ |
| KLK5 | Human | 5’- AGGGAGCAGTGGGTGGTTAT-3’ | 5’- ACCCTCACCAGGTCTCACTT -3’ |
|  | Mouse | 5’- AGACTGCCAAAAGGATGCAC -3’ | 5’- CTGCTGCCCAGACTCATAGA -3’ |
| IVL | Human | 5’- GCCTCAGCCTTACTGTGAGT -3’ | 5’- GAGCTCCTGACTGAGGGCA -3’ |
|  | Mouse | 5’- CCCTCCTGTGAGTTTGTTT -3’ | 5’- GTGAAGACCTGGCATTGTG -3’ |
| IL1α | Human | 5’- GTCAGCAAAGAAGTCAAGATGGC -3’ | 5’- CATGGAGTGGGCCATAGCTT -3’ |
|  | Mouse | 5’- TGAAACGTCAAAGATGTCCAA -3’ | 5’- AGGTGTAAGGTGCTGATCTGG -3’ |
| TAC1 | Human | 5’- GGTACGACAGCGACCAGATCA -3’ | 5’- AGCATCCCGTTTGCCCATTA -3’ |
|  | Mouse | 5’- AAAGAACTGCTGAGGCTTGG -3’ | 5’- TTTTCTCGTTTCCACTCAACTG -3’ |
| IL23 | Human | 5’- ATTTTCACAGGGGAGCCTTC -3’ | 5’- TTTTGAAGCGGAGAAGGAGA -3’ |
|  | Mouse | 5’- AGCAGCTCTCTCGGAATCTCT -3’ | 5’- AGGCTCCCCTTTGAAGATGT -3’ |
| IL1β | Human | 5’- ACCAAACCTCTTCGAGGCAC -3’ | 5’- AGCCATCATTTCACTGGCGA -3’ |
|  | Mouse | 5’- CAACCAACAAGTGATATTCTCCATG -3’ | 5’- GATCCACACTCTCCAGCTGCA -3’ |
| IL22 | Human | 5’- CCAGCCTTATATGCAGGAGG -3’ | 5’- TTTCAGCTTTGCTCTGGTCA -3’ |
|  | Mouse | 5’- ACATGCAGGAGGTGGTACCTTT -3’ | 5’- ATCGCCTTGATCTCTCCACTCT -3’ |
| KRT10 | Human | 5’- CAGTCCCAACTGGCCTTGAA -3’ | 5’- GGGCCTGAATCTGTGAGAGC -3’ |
|  | Mouse | 5’- CCACAGAAATCGACAGCAAC -3’ | 5’- TGGCTTTGAATCTGGGAGAG -3’ |
| IL20 | Human | 5’- ATCAGCAGCCTCGCCAATTCC -3’ | 5’- TCCTCCTATTCTGCTTCCTCCATCC -3’ |
|  | Mouse | 5’- GATAGGTGCTGCTTCCTTCG -3’ | 5’- CATTGCTTCTTCCCCACAAT -3’ |
| KRT6 | Human | 5’- ATGAAATCAACAAGCGCACA -3’ | 5’- TGTCTGAGATGTGGGTCTGC -3’ |
|  | Mouse | 5’- ACAACAACCGTAGCCTGGAC -3’ | 5’- TCTCAGCAATCTCCTGCTTG -3’ |
| IL17A | Human | 5’- CCCCAGTTGATTGGAAGAAA -3’ | 5’- GAGGACCTTTTGGGATTGGT -3’ |
|  | Mouse | 5’- GCTCCAGAAGGCCCTCAGA -3’ | 5’- AGCTTTCCCTCCGCATTGA -3’ |
| Cxcl3 | Human | 5’- CCACACTCAAGAATGGGAAGA -3’ | 5’- TCTCTCCTGTCAGTTGGTGCT -3’ |
|  | Mouse | 5’- TGGTCAAGAAGTTTGCCTCA -3’ | 5’- GGATGGATCGCTTTTCTCTG -3’ |
| S100A9 | Human | 5’- AGGGAGACAAGCACAAGCTGAAGA -3’ | 5’- TGTCCACAACCTCCTGCTCTTTGA -3’ |
|  | Mouse | 5’- GAAGGAATTCAGACAAATGG -3’ | 5’- ATCAACTTTGCCATCAGC -3’ |
| BD3 | Human | 5’- TTATTGCAGAGTCAGAGGCGG -3’ | 5’- CGAGCACTTGCCGATCTGTT -3’ |
|  | Mouse | 5’- TCAGATTGGCAGTTGTGGAG -3’ | 5’- GGGAGCACTTGTTTGCATTT-3’ |
| GAPDH | Human | 5’- AATGGGCAGCCGTTAGGAAA -3’ | 5’- GCGCCCAATACGACCAAATC -3’ |
|  | Mouse | 5’- TGCGACTTCAACAGCAACTC-3’ | 5’- CTTGCTCAGTGTCCTTGCTG -3’ |

**Supplementary Table S1:** List of primer sequences used in the study.
